# Cooling the Cochlea: Slowing Down Metabolism May Be a Way of Protecting Hearing from Surgical Trauma

**DOI:** 10.3390/medicina59071187

**Published:** 2023-06-22

**Authors:** Andrew Bell, W. Wiktor Jedrzejczak

**Affiliations:** 1 John Curtin School of Medical Research, The Australian National University, Canberra, ACT 2601, Australia; 2 Institute of Physiology and Pathology of Hearing, ul. Mochnackiego 10, 02-042 Warsaw, Poland; w.jedrzejczak@ifps.org.pl; 3 World Hearing Centre, ul. Mokra 17, 05-830 Kajetany, Poland

**Keywords:** hearing, extended high frequency, temperature

## Abstract

*Background and Objectives*: This narrative review of the literature explores the effect of body temperature on hearing. In particular, its focus is on extended high frequency (EHF) hearing—the range beyond the standard audiometric limit of 8 kHz. Such high frequencies are the first to be affected by noise-induced hearing loss, and so monitoring them can provide an early warning sign of incipient damage. *Materials and Methods*: This review builds on a personal literature database of 216 references covering the general topic of EHF hearing; the procedure was to then identify papers related to whole-body or cochlear cooling. A starting point was the paper by Munjal et al. who in 2013 reported changes of up to 15–30 dB in the EHF thresholds of subjects who had undergone cardiopulmonary bypass (CBP) surgery, which typically involves mild to moderate hypothermia—cooling of the blood—to reduce cellular oxygen demand and minimise tissue damage. *Results*: Reviewing the surrounding literature, we find that although CBP surgery by itself can impair hearing thresholds, lower body and cochlear temperatures in general provide neuroprotective effects. A connection between hearing loss and CBP surgery has been periodically documented, but the mechanism behind it has yet to be conclusively identified. *Conclusions*: The observations reviewed here tend to confirm the otoprotective effects of cooling. We consider that the high sensitivity of EHF thresholds to temperature is a major factor that has not been sufficiently recognised, although it has important implications for otological research and practice. Two important inferences are that, first, monitoring EHF thresholds might have considerable value in audiology, and, second, that lowering temperature of the cochlea during cochlear implantation might provide substantially better hearing preservation, as some researchers have already suggested.

## 1. Introduction

Extended high frequency (EHF) hearing is concerned with hearing beyond the range encompassed by the traditional audiogram. While audiologists typically measure hearing thresholds from 0.125 to 8 kHz, human hearing continues up to 20 kHz or more. Measurement and calibration at frequencies between 8 and 20 kHz is more difficult, while speech discrimination is largely unaffected by losses in this band. Nevertheless, auditory researchers are now becoming aware that these frequencies are important, mainly because they are the first frequencies to be affected by hearing loss, which progressively moves to lower frequencies as it becomes worse [1]. Thus, loss in the EHF range is an important warning sign of impending hearing deterioration. For a useful review of EHF hearing, the reader is referred to [2].

EHFs have been of interest for a number of decades and publications can be found dating back to the 1970′s (e.g., [3]). However, in recent years there has been a significant increase in interest. For example, when the search term ‘extended high frequency hearing’ is put into PubMed, 15 papers were found for 2012 while there were 53 for 2022 (search made 20 January 2023). Although the topic is now extensive, there is still no explanation of why hearing loss is first affected in the EHF range and what the mechanism is underlying the loss. This contribution does not seek to present a complete synthesis of EHF hearing and all its relevant factors—it is presently too early to undertake such an ambitious task. However, among the diverse findings in the literature, we were startled by one remarkable result. In 2013, when 30 patients underwent cardiopulmonary bypass (CBP) surgery, they experienced a hearing loss of 15–30 dB at 8 kHz or higher, even though lower frequencies were largely preserved [4].

This narrative review explores this curious finding in more detail, and explores several associated factors that appear important in EHF hearing. Recognising that CBP surgery typically involves extracorporeal circulation (bypass and cooling of the blood), we identify one or two factors—notably reduced body temperature and anesthesia—which appear to be key. The associated literature supports the importance of these factors, and here we set out some relevant findings. These contributory factors have only recently begun to be recognised in the auditory literature, and, interestingly, recent experiments indicate that low body temperature may well be otoprotective. A recent review by Péus et al. [5] identified 10 clinical papers which directly showed that cooling of the ear is protective against inner ear damage and disease. However, the literature extends far more widely than this small number, and here we add circumstantial, otoacoustic, and experimental studies which allow a more comprehensive picture to emerge. The particular focus here concerns losses to extended-high frequency hearing (8–20 kHz). The Péus et al. work confined itself to the otoprotective properties of hypothermia, and used a limited number of search terms involving hearing loss and cooling interventions. (The paper lists the search terms used, but prefaces them with “e.g.,” when the authors probably meant “i.e.,”). The authors initially identified some 420 papers, but cut them down to just 10 by focusing only on those involving clinical approaches aimed at ear protection via cooling, although the exclusion criteria they used were not made clear. We are simply told that fundamental studies were excluded, as were those looking at general neuroprotective effects and circumstantial evidence derived from CBP surgery. Péus et al. filled out eligibility forms designed in accordance with the review study inclusion criteria, and consulted with each other, but the way by which the final 10 papers were selected from the penultimate 33 studies is not evident from the flow chart provided. It is clear, however, that there are many more unexplored dimensions to this story, and this is what we aim to elucidate here.

## 2. Materials and Methods

Here, based on an initial personal database of 216 publications on EHF, we consider additional factors such as whole-body cooling and the results of otoacoustic measures which together point to the behaviour of the ear under conditions of lowered temperature. We emphasise that our review is in the form of an exploratory narrative, not a complete systematic review, but nevertheless we believe that the underlying pattern we uncover could be helpful. Our approach has been to examine the literature by starting with earlier reports on cardiovascular bypass (CBP) surgery, where the blood circulation is cooled, and look to see what other reports relate to the conclusion that temperature is a major factor in preserving the cochlea against insult.

Our examination of the literature leads us to agree with Péus et al. [5] that cooling of the ear is likely to be otoprotective. Our conclusion, based on what we admit is circumstantial evidence, is that hypothermia of the cochlea is promising, and it may open the way to a number of treatments that could be used to protect against hearing trauma, including cochlear implantation itself. We also point to indications that temperature may be a key factor underlying the generation of OAEs, and perhaps experimenters or clinicians involved in making OAE measurements should ensure that the body temperature of their subjects is checked, as even small variations might have big effects at high frequencies.

## 3. Emergence of the Temperature Factor

It is rather remarkable that, as related by the circumscribed review of Péus et al., the otoprotective effect of cryotherapy has been investigated since the 1980s, but, with few exceptions, local cooling in otorhinolaryngology has not been actively studied. The field has been left to just a handful of research groups. This contrasts with established clinical practice where thermal manipulation of the inner ear, such as with Barany’s caloric test, has become an established procedure in the diagnosis of the vestibular organ [6,7]. At the same time, the neuroprotective effects of cryotherapy have been routinely used in intensive care—to protect the brains of concussed or oxygen-deprived patients and during brain surgery, for example [8].

As mentioned earlier, the interest of scientists and clinicians in EHF is increasing. However, the literature is complex, and considerably more progress is needed before the picture becomes clear. However, as related above, among the diversity, there was a particularly intriguing clue: the 2013 report by Munjal et al. on 30 subjects, average age 61 years, who underwent CBP [4].

The EHF thresholds of these subjects were measured 1 day before and 2 weeks after surgery, and, surprisingly, the authors report that the subjects suffered an average hearing loss in the EHF range (10, 12, and 16 kHz) of some 3 dB, with the loss being greatest (5–6 dB) at the highest frequency. Losses were also measured at lower frequencies, although they were considerably lower, generally a fraction of a decibel. More seriously, however, 13 of the 30 subjects experienced an EHF loss of more than 15 dB, while 6 subjects suffered losses of more than 30 dB. More broadly, 19 of the 30 subjects could be identified as having had changes in cochlear function 2 weeks after surgery.

It is clear that something about the surgery itself is to blame, with 6 patients immediately reporting troublesome hearing loss after the operation. However, why should this loss be concentrated at the higher frequencies?

Reports of hearing loss after CBP surgery have been periodically reported for some decades, and Munjal et al. include them in their reference list. Curiously, the earliest reports refer to sudden, unilateral hearing loss, and various explanations have been offered. A lot happens during a protracted CBP operation, and the effects of anesthesia, ototoxic drugs, microembolisms, and reduced blood supply to the cochlea have been put forward as explanations [4] (p. 4). The neuroprotective role of cooling in general has been well studied [9], but the exact mechanism operating in the cochlea remains unclear. However, more recent work, with larger sample sizes and use of control groups, has shown that there are in fact more general, bilateral hearing losses after CBP surgery, and these studies provide additional insight.

**Figure 1:**
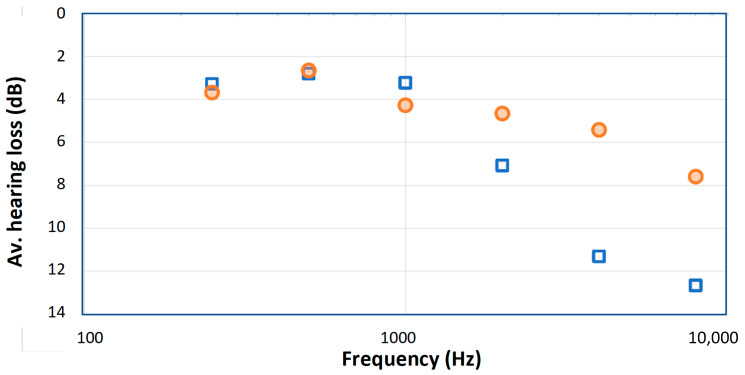
Average hearing losses after coronary graft surgery for two groups of patients reported by Aytacoglu et al. [10]: those who underwent bypass surgery involving cooling of the blood to 28 °C (blue squares) and those who remained normothermic (orange circles). Note that the bypass group suffered greater hearing loss as measured 3 days after surgery, indicating that the bypass procedure somehow created extra hearing damage, and that the losses were greatest at the highest frequencies.

Prominent among these more strictly controlled studies is work in 2006 by Aytacoglu et al. on two groups of patients [10]. One group underwent CBP surgery with moderate hypothermia (28 °C, *n* = 20), while the other group underwent a similar operation without the use of bypass (normothermic, *n* = 17). All operations were carried out by the same surgeon. The authors measured hearing thresholds from 0.25 to 8 kHz immediately before and on the third day following surgery and compared the results using a blinded analysis. Although the highest frequency tested was 8 kHz, the lower limit of our field of interest, the data is detailed and was published in tabular form so that it is a straightforward matter to extract the data and check for trends. Comparison between the hypothermic group and the normothermic group is shown in Figure 1.

Figure 1 makes clear that there is a distinct difference between the hypothermic and normothermic groups, and that the losses are concentrated at the highest frequencies. These trends were not plotted in the original text, which focused on identifying the number of extreme cases (>10–20 dB) in each group. The work by Aytacoglu et al. confirms that bypass surgery poses a clear risk to hearing, over and above the risk due to heart surgery alone. The risk depends on the individual patient, and for some patients it is substantial and for others undetectable. The authors tried to minimise the use of ototoxic drugs, including nitrous oxide, a known risk to hearing, but the considerable loss in both groups at high frequencies indicates that heart surgery, particularly bypass surgery, carries a risk to hearing. The authors suggest several reasons for the hearing damage, the most likely ones being perfusion changes due to altered circulatory parameters and the ototoxic effects of anesthesia (a range of other possibilities are discussed by [11,12]). If blood supply to the cochlea is impaired, hypoxia and hearing loss are likely to follow. However, vital signs of all the patients were kept within normal limits, and the authors found that there were no statistically significant correlation between hearing loss and common surgical parameters. This suggests that hypothermia may have been one of the factors that caused hearing loss. The authors’ conclusion is that “our findings [indicate] some adverse effects happening to the cochlea” during bypass surgery [10] (p. 256). However, what exactly are those factors?

## 4. Does Cooling Damage or Protect Hearing?

The findings outlined so far are intriguing, although it is not clear what the crucial factor (or factors) underlying hearing loss may be. It could be the cooling itself, it could be one of several ototoxic effects, or it could simply be the effect of blood viscosity, which increases at reduced temperatures and hence the circulation becomes less capable of supplying oxygen to the cochlea. More importantly, are these effects permanent or reversible, and is cooling always damaging? Significantly, there are now clues, supported by the review by Péus et al. [5] that, in general, cooling in fact tends to protect hearing. This implies that the poorer results after cooling evident in Figure 1 must be due to one or another unintended factors associated with the cooling regimen, but this is something that further research needs to clarify. As Tamames et al. point out, systemic hypothermia has a multitude of side-effects on the body, including impaired immune response, higher infection rates, and compromised vascular function [13], all of which present obstacles to clinical use. However, we believe the benefits of cooling may well outweigh the drawbacks, and is worth exploring further. The next section sets out the rationale for this statement in the context of the cochlea.

### Otoprotective Effects

The protective effect of low temperatures of animal tissues have been known for a considerable time, and forms the basis for cooling of the blood during cardiac surgery [8]. Studies on how the cochlea responds to lowered temperatures date from about the middle of last century, and all of them tend to indicate that, in experimental animals, cooling protects the cochlea from damage. Hypothermia produces a loss of cochlear sensitivity, which is greatest at high frequencies, although the loss is reversible once body temperature returns to normal [14]. During this cooling phase, the hair cells of animals are resistant to damage due to noise or ischemia [15].

Eshraghi et al. [16] undertook a research program to test whether mild or moderate hypothermia could protect the ear against the hearing loss which inevitably follows insertion of electrodes, such as happens with a cochlear implant. Their collective view was that cooling could reduce metabolic rate and oxygen consumption, decrease acidosis, suppress calcium influx into neurons, slow nitric oxide consumption, reduce glutamate excitotoxicity, and preserve permeability of the blood–brain barrier [16] (p. 926), all of which would be expected to preserve hearing under adverse conditions, and might be expected to reduce trauma during insertion of a cochlear implant. Working on rats, the group investigated auditory brainstem responses (ABRs) and distortion product otoacoustic emissions (DPOAEs) to measure cochlear performance after a mock electrode was inserted into the cochleas of the animals [17]. They found that hypothermia led to less functional loss immediately after surgery and, encouragingly, no progressive loss in the week following surgery. The same group continued their studies by showing that localised cooling of the cochlea (by 1–2 °C) could be produced by irrigating the rat ear canal with cold water, avoiding whole body cooling and its potential unwanted side-effects [18].

In their 2007 paper, Smith et al. [18] offer possible reasons for why the hearing losses illustrated in Figure 1 are worse under whole-body cooling conditions than when there is no cooling. They list impaired coagulation and immune function, longer hospitalisation, and increased incidence of wound infection and cardiac events [18] (p. 228). Clearly, these factors will need to be closely addressed in future work involving hypothermia. Encouragingly, however, the Eshraghi work emphasises that localised cooling has the potential to avoid most of these damaging side-effects while providing protection against cochlear trauma.

The Eshraghi group have continued to investigate localised cooling, and are one of the few groups strongly engaged in investigating the idea. In 2016 they published the results of placing a thermo¬electric probe in contact with the cochlea of the rat [13], reducing its temperature by 4–6 °C before they inserted an electrode-like implant. For 28 days the ABRs of a group of rats which received the cooling treatment were monitored and compared with those of another group in which the ears remained at normal temperature. The comparison showed that the hearing of the hypothermic rats, as measured by ABRs, was much less affected by the implantation at frequencies ranging from 0.5 kHz to 32 kHz.

**Figure 2:**
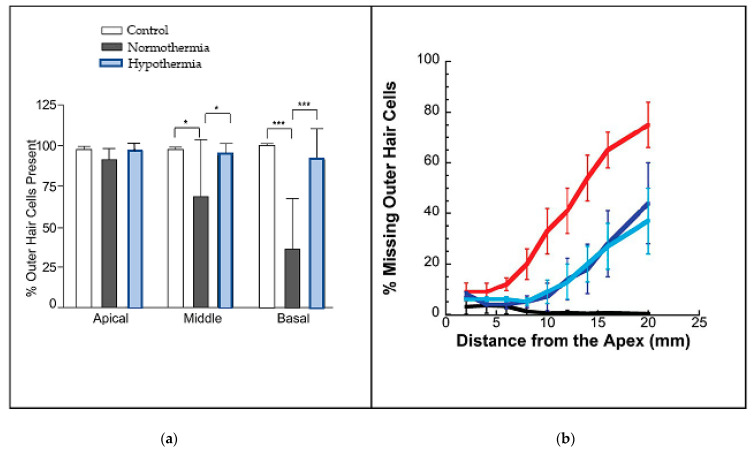
Cooling protects outer hair cells, particularly at high frequencies. (**a**) Percentage of outer hair cells still remaining when rats received an electrode-like implant (from [13], CC-BY-NC-ND; * is *p*<0.05; *** is *p*<0.005). (**b**) Percentage of missing OHCs in guinea pigs treated with cisplatin—when the ears of the animals underwent cooling, the loss of cells was much reduced (blue) compared to those animals whose ears were maintained at normal temperature (red). Black shows controls; light blue is with use of cooling bar; dark blue is with cold water. From [19], reproduced with permission of Wolters Kluwer.

Interestingly, the researchers extended their investigation to human temporal bones as well, finding that, when the thermoelectric probe was placed next to the round window niche, it reduced cochlear temperatures by 3–6 °C. The research program also involved histology, looking at how well cooling was able to preserve the number of outer hair cells (OHCs) present at the end of the 28 days. The findings were that the number of OHCs in the cooled cochleas was close to the number in the controls, whereas in the uncooled cochleas far fewer cells were counted. The results are reproduced in Figure 2a, which graphically shows how much protection cooling provides. Of particular interest, it is evident that protection applies most strongly at the base of the cochlea, where nearly 90% of the OHCs survived when cooled, compared to only about 30% survival if the cochlea was not cooled. At the apex, the OHCs are more robust and there was not much difference, while at the mid-regions the results were inbetween. These outcomes match the findings in Figure 1, where it is the high frequencies that are most susceptible to hearing loss from surgery, a result supported by Péus et al. [5].

Tamames et al. [13] conclude that mild to moderate hypothermia could be a useful therapy for aiding recovery from brain injuries and strokes and for improving neural function. They think that the neuroprotective mechanisms are probably multifactorial, but include suppression of free radicals and ototoxicity, less neural inflammation or disruption of the blood–brain barrier, and better regulation of early gene expression. To that end, there is one practically-oriented paper that provided a water-cooled pillow for patients being treated for sudden idiopathic hearing loss [20], although this was only able to decrease tympanic membrane temperature by little more than 1 °C. Nevertheless, even this small decrease was enough to provide better outcomes compared to a control group.

While findings like these are encouraging, for some reason the field has not been enthusiastic in taking the cooling idea further. So far as we are aware, there was no further research on the topic until a publication by the Spankovich group [19] reporting the results of irrigating the ear canals of guinea pigs with cool water or an electrically cooled ear bar. They found that, compared to controls, the cooling significantly reduced hearing loss and improved the survival of outer hair cells when the guinea pigs were dosed with cisplatin, an ototoxic compound used in chemotherapy. Figure 2b shows data telling a similar story to that in Figure 2a: the percentage of missing outer hair cells increased dramatically as the frequencies increased from the apex (the low-frequency end) to the base (the high-frequency end). Of particular interest, note that the loss was approximately halved in those animals provided with ear cooling.

Generalising so far, it seems clear that cooling of the ear is otoprotective, and that further investigations are called for in order to confirm this strategy. However, in following such a trail, there is one aspect that we wish to draw particular attention to—the role that otoacoustic emissions can play in monitoring the progress of cooling, especially the importance of using EHF measurements as a temperature probe, which, as we will see, are particularly sensitive to this parameter.

## 5. Otoacoustic Emissions

Otoacoustic emissions (OAEs) are faint sounds emitted by the cochlea in response to a click or tones and can be recorded with a microphone placed in the ear canal. Discovered by Kemp more than 40 years ago [21], OAEs provide a unique, noninvasive window into cochlea functioning and now comprise a huge literature [22,23].

Both distortion-product OAEs (DPOAEs) and click-evoked OAEs (CEOAEs) are, according to animal studies, highly temperature dependent [24]. In humans, a similar picture has emerged, although here the number of studies is limited. The first to study temperature effects on OAEs in humans were Veuillet et al. [25] who recorded TEOAEs of 5 children while they were in open-heart surgery involving cooling of their blood. The emissions underwent strong reductions, nearly 20 dB, during cooling, but the measurements they made were not frequency-specific, so they do not give information on EHF behaviour. Soon after, Seifert et al. [26] studied TEOAEs in 30 patients who were also undergoing open heart surgery; again, amplitudes soon disappeared into the noise floor after cooling began, but once more behaviour of different frequency bands was not of prime interest. Seifert later undertook cooling experiments with guinea pigs and came to similar conclusions [27], but again frequency sensitivity was not on the agenda. However, they did conclude that cooling affected not only the outer hair cells through reduction of the endocochlear potential, but probably also by having effects on cochlear efferents. They point out that various anesthetic agents affect cochlear blood flow, so that conclusions about the specific locus of hypothermia are likely to be inconclusive.

Turning to DPOAEs, Borin and Mendonca Cruz [28] studied these emissions in 18 patients undergoing cardiac bypass surgery involving hypothermia. They made measurements in frequency bands from 0.5 kHz to 5.5 kHz and found that the average drop in intensity during hypothermia ranged from 4–9 dB. Interestingly, however, the biggest changes were not observed at the highest frequencies. This is in marked contrast to Aydin et al. [29] who studied DPOAEs in 24 children who were also undergoing similar surgery and in this case the largest changes, up to 20 dB, took place at the highest frequencies (beyond 6 kHz). In another similar study, El Ganzoury et al. [30] reported that, in 40 children undergoing cardiac bypass surgery, their DPOAEs at 8.8 kHz were some 15 dB lower during moderate hypothermia (28–32 °C) than during mild hypothermia (33–37 °C), while at lower frequencies the changes were much less marked.

Of particular relevance, OAEs can be measured in the EHF range. Although most studies incorporate custom systems (e.g., [31,32]), there are also some commercial systems that provide this type of measurement [33]. Poling et al. [31] have shown that EHF OAEs provide useful information in monitoring hearing during chemotherapy. If proper calibration protocols are applied, EHF OAEs are repeatable [32] and measurable in all age groups [32,34]. Finally, it has recently been suggested that changes in OAEs in that range may be a good indicator of corresponding changes in EHF hearing thresholds [33,35]. Unfortunately there are no studies on effects of cooling on EHF OAEs so far.

## 6. General Discussion

From a consideration of the above, we can see that there are two major factors at play. The first, and perhaps the easiest to establish, is that hypothermia generally reduces the amplitudes of OAEs [36]. This is not greatly surprising, since it has been known for some time that OAEs depend on the metabolic activity of OHCs [22]. In particular, it has been reported that prestin, the outer hair cell motor protein, is highly temperature dependent [37], implying that OAE strength is likely to be a good tool for monitoring cochlear temperature.

The other important perspective is the indication that hypothermia is not just neuroprotective but otoprotective as well. Cryotherapy tends to increase tolerance to ischemia, reduce sensitivity to toxic chemicals, and reduce hearing loss in response to acoustic trauma. That is, following hypothermia, the cochlea will bounce back and will be more likely to regain its previous function than if the cooling was not applied, which is an important consideration for those undergoing cochlear implantation in which physical damage is apt to occur.

However, the best way of cooling the cochlea is presently unknown. Clearly, localised cooling is the only practical option, as whole-body cooling is complicated and carries severe risks. Head and neck cooling, as used by Hato et al. [20], appears to be too inefficient to be useful. Stanford et al. [19] are investigating the irrigation of the ear canal with cool water, as well as by inserting cool “ear bars” into the ear canal, and these approaches seem to be more promising. There may be better ways of providing localised cooling to the cochlea, and all of these possibilities require investigation. However the fact remains that, as of 2023, cryotherapy has not yet been attempted in humans.

The focus of the Stanford work is to avoid damage to hearing due to ototoxic materials, primarily cisplatin and similar therapeutic chemicals used for cancer therapies. However, another promising field is that of cochlear implantation. It is now more common for those undergoing hearing related surgeries to have “partial hearing”, in which they require a cochlear implant for receiving high frequencies, but still have residual hearing at low frequencies (below 1 kHz) and can still use those frequencies for natural hearing (with or without a conventional hearing aid) [38]. Making use of residual hearing as well as an implant provides better communication abilities than with just an implant alone. In such cases, we expect that a strategy of making use of hypothermia during implantation is worth investigating. Additionally, since there is some functional hearing at low frequencies (such as 0.5 kHz), there is scope for the effects of cochlear implantation (with cooling) to be constantly monitored via low-frequency OAEs [39]. Of course, there are other possible avenues for monitoring hearing status during surgery, of which perhaps electrocochleography is the most well known [40]. Monitoring of ABRs in humans also has an important role to play [41]. We do not wish to underplay the importance of these and other electrically based techniques, but for reasons of space and our own major focus on otoacoustics, we choose not to enter into a more extended discussion.

Nevertheless, for all the positive indicators for the effectiveness of cooling, there are still many unknowns. Perhaps the most significant, as pointed out by Péus et al. [5], is that the degree of cooling required to provide useful benefits has not yet been determined. As they put it, mild local hypothermia (4–6 °C) may prove tolerable but may not be enough to achieve effective otoprotection.

## 7. Conclusions

There are two useful conclusions from this narrative review. Firstly, that monitoring OAEs offers one way of keeping track of the temperature inside the cochlea. This applies particularly to OAEs in the extended high frequency region, which are particularly sensitive to temperature. The second is that hypothermia is otoprotective—that it protects the cochlea from mechanical insult and enables it to rebound to normal functioning after the hypothermia has been removed.

Both these conclusions require careful validation in order for progress to be made, but there appears to be considerable potential for benefitting patients undergoing cochlear surgery, notably those receiving cochlear implants and who also have residual low-frequency hearing.

## Data Availability

The data plotted in Figure 1 is available in [10].
